# Copy number variants of *ABCF1*, *IL17REL*, and *FCGR3A* are associated with the risk of gout

**DOI:** 10.1007/s13238-017-0401-y

**Published:** 2017-04-12

**Authors:** Zheng Dong, Yuan Li, Jingru Zhou, Shuai Jiang, Yi Wang, Yulin Chen, Dongbao Zhao, Chengde Yang, Qiaoxia Qian, Yanyun Ma, Hongjun He, Hengdong Ji, Yajun Yang, Xiaofeng Wang, Xia Xu, Yafei Pang, Hejian Zou, Li Jin, Feng Zhang, Jiucun Wang

**Affiliations:** 1 State Key Laboratory of Genetic Engineering, Collaborative Innovation Center for Genetics and Development, School of Life Sciences, Fudan University, Shanghai, 200433 China; 2 Division of Rheumatology and Immunology, Changhai Hospital, Shanghai, 200433 China; 3 Division of Rheumatology, Ruijin Hospital, Shanghai Jiaotong University School of Medicine, Shanghai, 200025 China; 4 Division of Rheumatology, Taixing People’s Hospital, Taixing, 225499 China; 5 Division of Rheumatology, Taizhou People’s Hospital, Taizhou, 225300 China; 6 Fudan-Taizhou Institute of Health Sciences, Taizhou, 225316 China; 7 Division of Rheumatology, Huashan Hospital, Fudan University, Shanghai, 200040 China; 8 Institute of Rheumatology, Immunology and Allergy, Fudan University, Shanghai, 200040 China

**Dear Editor,**

Gout is usually characterized by recurrent flares of acute inflammatory arthritis and the crystallization of urate in tissues and joints (Dong et al., 2017). The formation of monosodium urate crystals (MSU) is due to the elevated uric acid levels in the blood (Yang et al., 2016). But only 10% of individuals with hyperuricemia develop gout, suggesting that certain people are at a higher risk of developing MSU crystals (Merriman and Dalbeth, 2011) or initiating of the acute inflammatory response than others. This phenomenon is likely explained by the complex pathogenic process of inflammation and innate immunity in the development of gout since the MSU crystal-mediated inflammatory pathways have been reported to associate with many immunological processes (Behrens et al., 2008). The above results suggest that inflammation- and immunity-related factors can regulate the development of gout, particularly the progression from hyperuricemia to gout.

Recent genome-wide association studies (GWAS) identified some single nucleotide polymorphisms (SNPs) that were associated with the risk of gout (Kottgen et al., 2013). However, the common SNPs only explained part of the heritability of gout, and copy number variants (CNVs), an important source of genetic diversity in humans, have been shown to play a critical role in the genetic susceptibility to multiple rheumatic diseases (Zhang et al., 2009). Therefore, CNVs might be a new source for the genetic variants associated with the pathogenesis of gout. However, CNV studies on gout have not been reported, making it necessary to explore the pathogenesis of gout through the copy number variants in the inflammation- and immunity-related genes.

In this study, we aimed to identify the gout-associated CNVs in a Chinese population using a three-step approach. The entire analysis strategy, from genome-wide CNV discovery to gout-associated CNV identification to CNV validation, was illustrated in Fig. S1. Using this strategy, we provided the first evidence that CNVs could influence the pathogenesis of gout, and identified three novel genes, *ABCF1*, *IL17REL* and *FCGR3A*, which are associated with the risk of gout.

Based on a genome-wide analysis of CNVs, a total of 1,901 CNVs were identified in 22 patients with inflammation- and immunity-related rheumatic disorders (inclunding gout, ankylosing spondylitis, systemic lupus erythematosus, and systemic sclerosis) compared to the controls by using Agilent SurePrint G3 Human 1 × 1 M comparative genomic hybridization (CGH) microarray. After they were filtered according to their frequency (>5% and ≤50%), genome location (in gene region), gene function (related with inflammation and immunity), reliability (at least five consecutive probes) and previous studies for these CNVs, 48 gene regions with CNVs were selected (Table S1). To identify candidate gout-associated CNVs, the above pre-selected CNVs were further tested in 46 gout patients and 47 healthy Chinese individuals using the NimbleGen CGH Arrays (12 × 270 K), which is a high-density CGH assay, designed and made by Roche. Five CNVs appeared in more than 5% of the subjects, and located at a chromosome region that contained exons for inflammation- and immunity-related genes (*ABCF1*, *IL17REL*, *FCGR3A*, *DPCR1*, and *DEFA10P*), and were identified to be candidate CNVs associated with gout.

To validate the significance of the CNVs, the above five candidate CNVs (Table S2) were tested in an additional 1,274 Chinese individuals (576 gout patients and 698 control subjects, Tables S3 and S4) using AccuCopy^TM^ (Du et al., 2012). The distributions of three CNVs in *ABCF1*, *IL17REL*, and *FCGR3A* were significantly different between the gout patients and the controls according to Fisher’s exact test, with *P* values of 0.018, 0.021 and 0.022, respectively (Tables S5 and S6). After correcting for multiple comparisons, the distributions of these CNVs were still significantly different (all *P* = 0.037) (Table S5). In addition, to avoid the heterogeneity of gender and age, deviance analysis for the logistic regression model adjusted for gender and age also showed that the CNVs in *IL17REL* and *FCGR3A* were significantly different between gout patients and control subjects (*P* = 0.002 and 0.038, respectively). The other two CNVs in the *DPCR1* and *DEFA10P* genes were not associated with the risk of gout in the Chinese population (*P* = 0.328 and 0.221, respectively).

The *ABCF1* gene encodes a superfamily member of the ATP-binding cassette (ABC) transporters. The ABC transporter gene family transports various substrate molecules across the extra- and intra-cellular membranes and has an important contribution to the mechanisms of cellular defense (Hoh et al., 2014). ABCF1 protein has been reported to both regulate and be regulated by TNF and may regulate the translation of inflammatory cytokines (Powell et al., 2013). In this study, we first found that the copy number variant of the *ABCF1* gene may play an important role in the pathogenesis of gout and its high copy number (CN > 2) strongly increased the risk of gout (Odds ratio (OR) 4.33, *P* = 0.007), particularly in males (OR 5.70, *P* = 0.002) (Tables 1, S5, and S7), which suggests a potential contribution of *ABCF1* in the inflammatory process of gout. To the best of our knowledge, the high copy number of *ABCF1* had the strongest risk effect on the pathogenesis of gout in males (OR 5.70, *P* = 0.002) than any other genetic factors that had previously been reported. And the effects of the CNV in the *ABCF1* on the risk of gout were also significant after adjusting for gender and age (*P* = 0.004) (Table 1). In the male subgroup, the CNV also influenced the risk of gout after adjusting for age (*P* = 0.003).

**Table 1:** Copy number variants in genes influenced the risk of gout

CNV region			OR	P*	*P* ^#^
*ABCF1*	CN > 2 vs. CN = 2	Male	5.70	0.002	0.003
		Female	NA	1.000	0.513
		Total	4.33	0.007	0.004
*IL17REL*	CN < 2 vs. CN = 2	Male	0.15^$^	0.009	0.003
		Female	1.93	0.506	0.568
		Total	0.24	0.076	0.021
*FCGR3A*	CN > 2 vs. CN = 2	Male	0.61	0.017	0.024
		Female	0.95	1.000	0.968
		Total	0.60	0.011	0.042

* P values were calculated by Fisher’s exact test. *P* ^#^ means *P* values were calculated by deviance analysis for logistic regression model after adjusted for gender and age. ^$^ OR = 0.15 was calculated when the low copy number in case was defined as 1. CN is the abbreviation of copy number. OR is the abbreviation of odds ratio. CN = 2 was treated as referent group

A previous GWAS study found that the *IL17REL* gene was associated with ulcerative colitis, a form of inflammatory bowel disease (Franke et al., 2010). IL17REL may bind specific IL17 cytokines and function as a potential negative regulator of other IL17R subfamilies (Wu et al., 2011). The interleukin 17 receptor (IL17R) family has been shown to affect the inflammatory/immune responses and influence the development of autoimmune disorders. In this study, we found that it may play an important role in the pathogenesis of gout (Tables 1, S5, and S7). The low copy number (CN < 2) of the *IL17REL* gene was a protective factor for gout (OR 0.24, *P* = 0.076) and might be the strongest protective effect against the development of gout in males than any other genetic factors that had previously been reported (OR 0.15, *P* = 0.009). After adjusting for gender and age, the effects of the CNV in the *IL17REL* on the risk of gout was also significant (*P* = 0.021) (Table 1), especially in the male subgroup (*P* = 0.003).

In addition, the CNVs in the *FCGR3A* gene were reported to be associated with a number of disorders, such as autoantibody-positive rheumatoid arthritis (Robinson et al., 2012) and systemic lupus erythematosus (Chen et al., 2014a), suggesting the important contributions of the *FCGR3A* copy number variants on the pathogenesis of diseases. Our present study first identified that CNVs in the *FCGR3A* gene were associated with the risk of gout (Tables 1, S5, and S7), and the CNV in the locus showed different distributions of frequency between gout patients and healthy subjects, particularly the frequency of the high *FCGR3A* copy number variant (7.46% of gout patients and 11.89% of controls). The high copy number (CN > 2) of the *FCGR3A* gene was also a protective factor for gout without (OR 0.60, *P* = 0.011) or with adjustment gender and age (*P* = 0.024) (Table 1).

Previous studies reported that CNVs can modify gene expression level and lead to consequent phenotypes by changing gene dosage (Zhang et al., 2009; Dong and Wang, 2015). To further validate the above results, the present study randomly selected the RNAs from 42 male gout patients and 46 healthy males and tested the differences in the mRNA levels of the candidate genes. All of three candidate genes, *ABCF1*, *IL17REL* and *FCGR3A*, showed a significant difference in mRNA expression according to Student’s *t*-test, with *P* values of 4.09 × 10^−9^, 0.014 and 0.011, respectively (Fig. 1). Interestingly, the higher percentage of *ABCF1* duplication in the cases was consistent with the higher expression in these subjects, indicating that the positive correlation between gene dosage and mRNA expression. The high expression of *ABCF1* may regulate the translation of inflammatory cytokines and thus affect the development of gout. The distributions of candidate CNVs in *FCGR3A* and *IL17REL* genes between the gout patients and the controls were also consistent with the distributions of mRNA levels between them, supporting the positive correlation and suggesting the two genes may influence the process of inflammation and immunity leading to gout via dosage effect. Previous studies also found that a gene-dosage effect of *FCGR3A* caused by CNVs was correlated with FcγRIIIa expression in NK cells (Breunis et al., 2009; Chen et al., 2014a). In addition, by analyzing Geuvadis data and Genotype-Tissue Expression Data Portal, many expression quantitative trait loci (eQTL) for the single/multi-tissues in European population or other populations’ samples were also found in the candidate CNVs of *ABCF1* and *FCGR3A*, suggesting the variants in those CNV regions played an important role in mRNA expression. CNV could influence the genotype of eQTL and the dosage of gene, leading to the change of transcriptional levels. All these results suggested that the candidate CNVs might influence the risk of gout by changing the mRNA expression of these genes.

**Figure 1:**
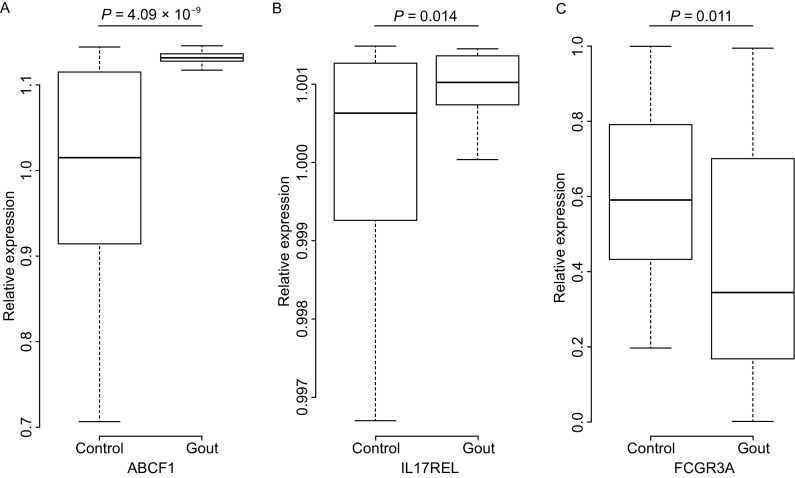
**Messenger RNA levels of candidate genes in blood samples from gout patients and healthy individuals**. (A) *ABCF1* gene; (B) *IL17REL* gene; (C) *FCGR3A* gene. SYBR Green-based quantitative polymerase chain reaction (qPCR) was used to test the transcription levels of candidate genes. Data of relative expression were analyzed by Student’s *t*-test. Data are illustrated as boxplots. Upper edge and lower edge of box represents the 75th percentiles and 25th percentiles, respectively. The line inside the boxes represents the median of data

In conclusion, the present study identified three novel genes, *ABCF1*, *IL17REL* and *FCGR3A*, associated with the pathogenesis of gout and showed, for the first time, that copy number variants can influence the risk of gout. The high copy number variant in the *ABCF1* gene was the strongest risk factor for gout (OR = 5.70), while a low copy number variant in the *IL17REL* gene was the strongest protective factor against gout (OR = 0.15) compared to other genetic factors that had previously been reported. In addition, our study indicates that CNV analyses could be a good way to explore the genetic variants associated with the risk of gout, which is helpful to understand the pathogenesis of gout and suggest its potential contribution for prediction, prevention, and treatment of gout.

## Electronic supplementary material

Below is the link to the electronic supplementary material.

Supplementary material 1 (PDF 844 kb)

Supplementary material 2 (PDF 97 kb)
