# Supplementary material for: Copy number variants of ABCF1, IL17REL, and FCGR3A are associated with the risk of gout
Source: Protein Cell. 2017 Apr 12;8(6):467–70. doi: 10.1007/s13238-017-0401-y (PMC5445030; doi:10.1007/s13238-017-0401-y)
Supplement: Supplementary file 1 — Supplementary material 1 (PDF 844 kb) [file 13238_2017_401_MOESM1_ESM.pdf]

Supplementary Materials

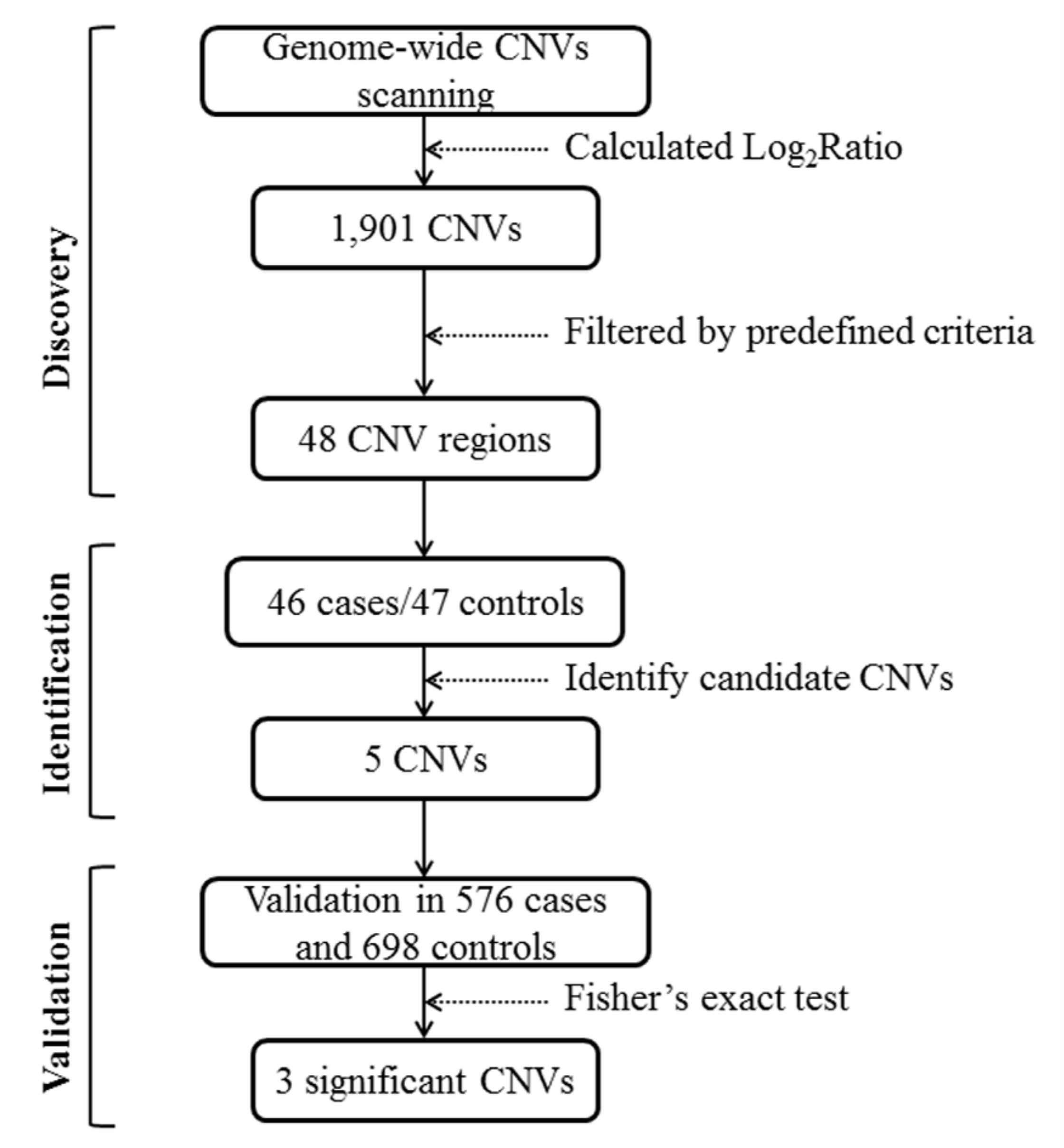

Fig. S1. The procedure from copy number variants (CNVs) discovery to validation. Briefly, Genome-wide analysis of CNVs was performed in 22 rheumatic disorders patients to find those inflammation and immunity related genes or regions with CNVs. After filtering out samples that did not satisfy the predefined criteria, those pre-selected CNVs were tested in 46 gout patients in contrast to 47 healthy Chinese individuals using aCGH to identify the candidate CNV loci. Finally, five candidate gout-associated CNVs were validated in a sample set containing 576 gout patients and 698 control subjects and three CNVs found to be significantly by Fisher's exact test.

Table S1. 48 gene regions with CNVs were discovered in discovery stage

Table S2. Primer sequences for CNVs in validation stage

Table S3. Characteristics of participants in this study separated by gender

Table S4. Characteristics of healthy participants in this study

Table S5. Copy number variants associated with the risk of gout

Table S6. Copy number for each individual with abnormal copy numbers

Table S7. Copy number variants associated with the risk of gout in subgroups of gender

Table S1. 48 gene regions with CNVs were discovered in discovery stage

**Genes**
CCL3L1
IRGM
IL-22
KLRC2
GSTM1
B2M
IL-17F
IL-21
IRF1
ZNF300
C6
MALL/NPHP1
TRAF3
KIR
CD44
CCL4/CCL4L1
PRKCH
MLL3
LCE Loci
PDPR
CDC42EP3
ALOX5AP
APOBEC3A/APOBEC3B
CCNG2/CXCL13

CRYBB2/ADRBK2
C4
CFHR3/CFHR1
TNIP1
METTL9
ITGB1
VPREB1
HLA
DEFA/DEFB
FCGR Locus
MGAM
TBX21
LCP2
B3GAT2
TLR7
MMP2
NUDT12
FcGRT
NCF1
IL-12B
TNFAIP3
IL17REL
HRH4
HP/HPR

Table S2. Primer sequences for CNVs in validation stage

CNVs	Chr.	Location (ref37)	Amplifed length (bp)	Primer 1	Primer 2
ABCF1	Chr6	30539178-30539267	118	TTGGAGAGCCAGCCCCATC	CTGTAACTGCCACCGCGATG
IL17REL	Chr22	50436717-50436825	137	TGGTCACTTGAGTGGGTGCTAGA	CACTGAGGCACTGGAGGTGCT
FCGR3A	Chr1	161514599-161514859	289	AGTCAGAATTATGATGAAAATACTTCCTGC	ACATAAGGGAAAGCCAGATTGGG
DPCR1	Chr6	30919077-30919176	128	GGAGAAAGGATAGCCAATGAGAAGG	GACACCACACCATCCTCAGCAG
DEFA10P	Chr8	6826414-6826618	233	TCCAACCCTCTAGACTGTGCAGC	AAAGAGAACGGTGGCAGTGAGG

Table S3. Characteristics of participants in this study separated by gender

Characteristics	Control	Gout
**Male**		
Number	599	503
Age	71.81 (6.50)	53.04 (14.53)
BMI	23.69 (2.74)	25.96 (4.31)
**Female**		
Number	99	26
Age	66.63 (7.50)	66.50 (17.95)
BMI	23.56 (3.22)	25.21 (4.03)
**Total**		
Number	698	576
Age	71.10 (6.94)	53.61 (15.14)
BMI	23.67 (2.80)	25.92 (4.29)

Data are shown as the mean (SD).

Table S4. Characteristics of healthy participants in this study

	**Male**	**Female**
Number	599	99
Height (cm)	164.09 (6.01)	152.94 (5.21)
Weight (kg)	63.81 (8.52)	55.15 (8.26)
Smoking rate	62.10%	7.07%
Serum Urate (umol/l)	311.01 (55.51)	263.58 (51.19)
total bilirubin (umol/l)	19.77 (7.84)	15.97 (5.92)
Glucose (mmol/L)	5.52 (1.59)	5.34 (1.40)
Cholesterol (mmol/L)	4.55 (0.86)	5.05 (1.07)
Triglyceride (mmol/L)	1.29 (0.72)	1.28 (0.57)
Creatinine (umol/L)	77.79 (15.56)	60.21 (16.90)
blood urea nitrogen (mmol/L)	5.76 (3.09)	5.20 (1.31)

Table S5. Copy number variants associated with the risk of gout

CNV region	CNV Position		CN < 2	CN = 2	CN > 2	*P**	P_{BH}
ABCF1	Chr6: 30,462,062-30,562,634	Case	11	551	14	0.018	0.037
		Control	12	682	4		
IL17REL	Chr22: 50,431,602-50,437,690	Case	2	571	3	0.021	0.037
		Control	10	688	0		
FCGR3A	Chr1: 161,481,292-161,539,013	Case	10	523	43	0.022	0.037
		Control	8	607	83		
DPCR1	Chr6: 30,917,090-30,919,612	Case	10	566	0	0.328	0.328
		Control	7	691	0		
DEFA10P	Chr8: 6,825,837-6,828,012	Case	158	420	0	0.221	0.276
		Control	169	529	0		

**P* values were calculated by Fisher's exact test. P_{BH} means *P* values adjusted for multiple comparisons correction using BH method. CN is the abbreviation of copy number.

Table S6. Copy number for each individual with abnormal copy numbers

Sample No.	Copy Numbers (CN)				
	IR17REL	***FCGR3A***	***DPCR1***	***DEFA10P***	***ABCF1***
Sample571	1	2	2	0	1
Sample566	1	2	2	0	2
Sample572	1	2	2	1	2
Sample595	1	2	2	2	1
Sample584	1	2	2	2	2
Sample1196	1	2	2	2	2
Sample1206	1	2	2	2	2
Sample1273	1	2	2	2	2
Sample586	1	3	2	1	2
Sample565	1	3	2	2	1
Sample1198	1	3	2	2	1
Sample564	1	3	2	2	2
Sample225	2	1	2	0	2
Sample605	2	1	2	0	2
Sample394	2	1	2	1	2
Sample561	2	1	2	1	2
Sample825	2	1	2	1	2
Sample826	2	1	2	1	2
Sample959	2	1	2	1	2
Sample9	2	1	2	2	2
Sample98	2	1	2	2	2
Sample101	2	1	2	2	2
Sample117	2	1	2	2	2
Sample349	2	1	2	2	2
Sample696	2	1	2	2	2
Sample895	2	1	2	2	2

Sample955	2	1	2	2	2
Sample986	2	1	2	2	2
Sample1235	2	1	2	2	2
Sample1266	2	1	2	2	2
Sample1212	2	2	1	1	2
Sample26	2	2	1	2	2
Sample236	2	2	1	2	2
Sample239	2	2	1	2	2
Sample371	2	2	1	2	2
Sample449	2	2	1	2	2
Sample681	2	2	1	2	2
Sample708	2	2	1	2	2
Sample718	2	2	1	2	2
Sample742	2	2	1	2	2
Sample800	2	2	1	2	2
Sample827	2	2	1	2	2
Sample876	2	2	1	2	2
Sample1006	2	2	1	2	2
Sample951	2	2	1	2	3
Sample10	2	2	2	0	2
Sample38	2	2	2	0	2
Sample194	2	2	2	0	2
Sample284	2	2	2	0	2
Sample322	2	2	2	0	2
Sample357	2	2	2	0	2
Sample395	2	2	2	0	2
Sample441	2	2	2	0	2
Sample469	2	2	2	0	2
Sample492	2	2	2	0	2

Sample724	2	2	2	0	2
Sample725	2	2	2	0	2
Sample745	2	2	2	0	2
Sample792	2	2	2	0	2
Sample890	2	2	2	0	2
Sample928	2	2	2	0	2
Sample1086	2	2	2	0	2
Sample1119	2	2	2	0	2
Sample1214	2	2	2	0	2
Sample590	2	2	2	1	1
Sample671	2	2	2	1	1
Sample686	2	2	2	1	1
Sample749	2	2	2	1	1
Sample6	2	2	2	1	2
Sample11	2	2	2	1	2
Sample23	2	2	2	1	2
Sample27	2	2	2	1	2
Sample43	2	2	2	1	2
Sample45	2	2	2	1	2
Sample47	2	2	2	1	2
Sample53	2	2	2	1	2
Sample54	2	2	2	1	2
Sample56	2	2	2	1	2
Sample66	2	2	2	1	2
Sample69	2	2	2	1	2
Sample72	2	2	2	1	2
Sample73	2	2	2	1	2
Sample74	2	2	2	1	2
Sample75	2	2	2	1	2

Sample81	2	2	2	1	2
Sample86	2	2	2	1	2
Sample90	2	2	2	1	2
Sample100	2	2	2	1	2
Sample104	2	2	2	1	2
Sample112	2	2	2	1	2
Sample113	2	2	2	1	2
Sample120	2	2	2	1	2
Sample123	2	2	2	1	2
Sample140	2	2	2	1	2
Sample143	2	2	2	1	2
Sample147	2	2	2	1	2
Sample152	2	2	2	1	2
Sample157	2	2	2	1	2
Sample163	2	2	2	1	2
Sample170	2	2	2	1	2
Sample182	2	2	2	1	2
Sample183	2	2	2	1	2
Sample187	2	2	2	1	2
Sample188	2	2	2	1	2
Sample192	2	2	2	1	2
Sample197	2	2	2	1	2
Sample201	2	2	2	1	2
Sample209	2	2	2	1	2
Sample210	2	2	2	1	2
Sample211	2	2	2	1	2
Sample212	2	2	2	1	2
Sample224	2	2	2	1	2
Sample227	2	2	2	1	2

Sample235	2	2	2	1	2
Sample240	2	2	2	1	2
Sample242	2	2	2	1	2
Sample247	2	2	2	1	2
Sample249	2	2	2	1	2
Sample256	2	2	2	1	2
Sample258	2	2	2	1	2
Sample263	2	2	2	1	2
Sample268	2	2	2	1	2
Sample273	2	2	2	1	2
Sample278	2	2	2	1	2
Sample286	2	2	2	1	2
Sample293	2	2	2	1	2
Sample297	2	2	2	1	2
Sample298	2	2	2	1	2
Sample307	2	2	2	1	2
Sample314	2	2	2	1	2
Sample319	2	2	2	1	2
Sample320	2	2	2	1	2
Sample323	2	2	2	1	2
Sample324	2	2	2	1	2
Sample325	2	2	2	1	2
Sample327	2	2	2	1	2
Sample334	2	2	2	1	2
Sample335	2	2	2	1	2
Sample340	2	2	2	1	2
Sample342	2	2	2	1	2
Sample350	2	2	2	1	2
Sample354	2	2	2	1	2

Sample356	2	2	2	1	2
Sample374	2	2	2	1	2
Sample375	2	2	2	1	2
Sample376	2	2	2	1	2
Sample380	2	2	2	1	2
Sample383	2	2	2	1	2
Sample388	2	2	2	1	2
Sample391	2	2	2	1	2
Sample397	2	2	2	1	2
Sample398	2	2	2	1	2
Sample401	2	2	2	1	2
Sample415	2	2	2	1	2
Sample422	2	2	2	1	2
Sample423	2	2	2	1	2
Sample428	2	2	2	1	2
Sample431	2	2	2	1	2
Sample440	2	2	2	1	2
Sample455	2	2	2	1	2
Sample464	2	2	2	1	2
Sample482	2	2	2	1	2
Sample486	2	2	2	1	2
Sample490	2	2	2	1	2
Sample493	2	2	2	1	2
Sample495	2	2	2	1	2
Sample500	2	2	2	1	2
Sample504	2	2	2	1	2
Sample505	2	2	2	1	2
Sample514	2	2	2	1	2
Sample516	2	2	2	1	2

Sample523	2	2	2	1	2
Sample526	2	2	2	1	2
Sample527	2	2	2	1	2
Sample531	2	2	2	1	2
Sample537	2	2	2	1	2
Sample545	2	2	2	1	2
Sample562	2	2	2	1	2
Sample588	2	2	2	1	2
Sample593	2	2	2	1	2
Sample601	2	2	2	1	2
Sample609	2	2	2	1	2
Sample612	2	2	2	1	2
Sample613	2	2	2	1	2
Sample622	2	2	2	1	2
Sample627	2	2	2	1	2
Sample636	2	2	2	1	2
Sample637	2	2	2	1	2
Sample649	2	2	2	1	2
Sample653	2	2	2	1	2
Sample658	2	2	2	1	2
Sample663	2	2	2	1	2
Sample664	2	2	2	1	2
Sample667	2	2	2	1	2
Sample682	2	2	2	1	2
Sample687	2	2	2	1	2
Sample688	2	2	2	1	2
Sample690	2	2	2	1	2
Sample695	2	2	2	1	2
Sample705	2	2	2	1	2

Sample709	2	2	2	1	2
Sample710	2	2	2	1	2
Sample713	2	2	2	1	2
Sample714	2	2	2	1	2
Sample716	2	2	2	1	2
Sample723	2	2	2	1	2
Sample729	2	2	2	1	2
Sample731	2	2	2	1	2
Sample738	2	2	2	1	2
Sample748	2	2	2	1	2
Sample756	2	2	2	1	2
Sample757	2	2	2	1	2
Sample760	2	2	2	1	2
Sample766	2	2	2	1	2
Sample773	2	2	2	1	2
Sample778	2	2	2	1	2
Sample785	2	2	2	1	2
Sample787	2	2	2	1	2
Sample788	2	2	2	1	2
Sample791	2	2	2	1	2
Sample793	2	2	2	1	2
Sample798	2	2	2	1	2
Sample806	2	2	2	1	2
Sample813	2	2	2	1	2
Sample819	2	2	2	1	2
Sample829	2	2	2	1	2
Sample830	2	2	2	1	2
Sample834	2	2	2	1	2
Sample837	2	2	2	1	2

Sample852	2	2	2	1	2
Sample858	2	2	2	1	2
Sample867	2	2	2	1	2
Sample870	2	2	2	1	2
Sample874	2	2	2	1	2
Sample877	2	2	2	1	2
Sample878	2	2	2	1	2
Sample881	2	2	2	1	2
Sample883	2	2	2	1	2
Sample887	2	2	2	1	2
Sample891	2	2	2	1	2
Sample893	2	2	2	1	2
Sample894	2	2	2	1	2
Sample897	2	2	2	1	2
Sample914	2	2	2	1	2
Sample915	2	2	2	1	2
Sample916	2	2	2	1	2
Sample917	2	2	2	1	2
Sample920	2	2	2	1	2
Sample923	2	2	2	1	2
Sample932	2	2	2	1	2
Sample935	2	2	2	1	2
Sample938	2	2	2	1	2
Sample950	2	2	2	1	2
Sample956	2	2	2	1	2
Sample960	2	2	2	1	2
Sample965	2	2	2	1	2
Sample968	2	2	2	1	2
Sample976	2	2	2	1	2

Sample984	2	2	2	1	2
Sample993	2	2	2	1	2
Sample999	2	2	2	1	2
Sample1002	2	2	2	1	2
Sample1003	2	2	2	1	2
Sample1010	2	2	2	1	2
Sample1013	2	2	2	1	2
Sample1015	2	2	2	1	2
Sample1026	2	2	2	1	2
Sample1027	2	2	2	1	2
Sample1029	2	2	2	1	2
Sample1032	2	2	2	1	2
Sample1033	2	2	2	1	2
Sample1042	2	2	2	1	2
Sample1057	2	2	2	1	2
Sample1058	2	2	2	1	2
Sample1068	2	2	2	1	2
Sample1072	2	2	2	1	2
Sample1073	2	2	2	1	2
Sample1074	2	2	2	1	2
Sample1079	2	2	2	1	2
Sample1081	2	2	2	1	2
Sample1085	2	2	2	1	2
Sample1089	2	2	2	1	2
Sample1090	2	2	2	1	2
Sample1099	2	2	2	1	2
Sample1100	2	2	2	1	2
Sample1102	2	2	2	1	2
Sample1103	2	2	2	1	2

Sample1107	2	2	2	1	2
Sample1111	2	2	2	1	2
Sample1118	2	2	2	1	2
Sample1122	2	2	2	1	2
Sample1123	2	2	2	1	2
Sample1129	2	2	2	1	2
Sample1132	2	2	2	1	2
Sample1144	2	2	2	1	2
Sample1147	2	2	2	1	2
Sample1152	2	2	2	1	2
Sample1162	2	2	2	1	2
Sample1166	2	2	2	1	2
Sample1170	2	2	2	1	2
Sample1193	2	2	2	1	2
Sample1197	2	2	2	1	2
Sample1201	2	2	2	1	2
Sample1202	2	2	2	1	2
Sample1209	2	2	2	1	2
Sample1216	2	2	2	1	2
Sample1218	2	2	2	1	2
Sample1221	2	2	2	1	2
Sample1229	2	2	2	1	2
Sample1231	2	2	2	1	2
Sample1238	2	2	2	1	2
Sample1240	2	2	2	1	2
Sample1242	2	2	2	1	2
Sample1244	2	2	2	1	2
Sample1248	2	2	2	1	2
Sample1252	2	2	2	1	2

Sample1253	2	2	2	1	2
Sample1255	2	2	2	1	2
Sample1256	2	2	2	1	2
Sample1259	2	2	2	1	2
Sample1265	2	2	2	1	2
Sample1270	2	2	2	1	2
Sample889	2	2	2	1	3
Sample944	2	2	2	1	3
Sample949	2	2	2	1	3
Sample1051	2	2	2	1	3
Sample472	2	2	2	2	1
Sample570	2	2	2	2	1
Sample582	2	2	2	2	1
Sample640	2	2	2	2	1
Sample646	2	2	2	2	1
Sample720	2	2	2	2	1
Sample759	2	2	2	2	1
Sample794	2	2	2	2	1
Sample1200	2	2	2	2	1
Sample1227	2	2	2	2	1
Sample110	2	2	2	2	3
Sample216	2	2	2	2	3
Sample282	2	2	2	2	3
Sample843	2	2	2	2	3
Sample855	2	2	2	2	3
Sample865	2	2	2	2	3
Sample879	2	2	2	2	3
Sample940	2	2	2	2	3
Sample942	2	2	2	2	3

Sample943	2	2	2	2	3
Sample1054	2	2	2	2	3
Sample1165	2	2	2	2	3
Sample456	2	3	1	1	2
Sample266	2	3	2	0	2
Sample366	2	3	2	0	2
Sample583	2	3	2	1	1
Sample1213	2	3	2	1	1
Sample89	2	3	2	1	2
Sample92	2	3	2	1	2
Sample96	2	3	2	1	2
Sample167	2	3	2	1	2
Sample173	2	3	2	1	2
Sample180	2	3	2	1	2
Sample290	2	3	2	1	2
Sample408	2	3	2	1	2
Sample461	2	3	2	1	2
Sample579	2	3	2	1	2
Sample596	2	3	2	1	2
Sample602	2	3	2	1	2
Sample610	2	3	2	1	2
Sample674	2	3	2	1	2
Sample919	2	3	2	1	2
Sample964	2	3	2	1	2
Sample1098	2	3	2	1	2
Sample1104	2	3	2	1	2
Sample1143	2	3	2	1	2
Sample1172	2	3	2	1	2
Sample1236	2	3	2	1	2

Sample866	2	3	2	1	3
Sample4	2	3	2	2	1
Sample805	2	3	2	2	1
Sample37	2	3	2	2	2
Sample49	2	3	2	2	2
Sample52	2	3	2	2	2
Sample76	2	3	2	2	2
Sample77	2	3	2	2	2
Sample87	2	3	2	2	2
Sample103	2	3	2	2	2
Sample108	2	3	2	2	2
Sample111	2	3	2	2	2
Sample127	2	3	2	2	2
Sample135	2	3	2	2	2
Sample156	2	3	2	2	2
Sample169	2	3	2	2	2
Sample171	2	3	2	2	2
Sample175	2	3	2	2	2
Sample177	2	3	2	2	2
Sample178	2	3	2	2	2
Sample198	2	3	2	2	2
Sample199	2	3	2	2	2
Sample213	2	3	2	2	2
Sample234	2	3	2	2	2
Sample311	2	3	2	2	2
Sample343	2	3	2	2	2
Sample403	2	3	2	2	2
Sample407	2	3	2	2	2
Sample410	2	3	2	2	2

Sample427	2	3	2	2	2
Sample442	2	3	2	2	2
Sample445	2	3	2	2	2
Sample458	2	3	2	2	2
Sample459	2	3	2	2	2
Sample465	2	3	2	2	2
Sample481	2	3	2	2	2
Sample511	2	3	2	2	2
Sample540	2	3	2	2	2
Sample546	2	3	2	2	2
Sample552	2	3	2	2	2
Sample555	2	3	2	2	2
Sample558	2	3	2	2	2
Sample569	2	3	2	2	2
Sample574	2	3	2	2	2
Sample576	2	3	2	2	2
Sample577	2	3	2	2	2
Sample581	2	3	2	2	2
Sample587	2	3	2	2	2
Sample597	2	3	2	2	2
Sample598	2	3	2	2	2
Sample641	2	3	2	2	2
Sample645	2	3	2	2	2
Sample655	2	3	2	2	2
Sample692	2	3	2	2	2
Sample726	2	3	2	2	2
Sample784	2	3	2	2	2
Sample790	2	3	2	2	2
Sample795	2	3	2	2	2

Sample796	2	3	2	2	2
Sample797	2	3	2	2	2
Sample810	2	3	2	2	2
Sample817	2	3	2	2	2
Sample824	2	3	2	2	2
Sample835	2	3	2	2	2
Sample846	2	3	2	2	2
Sample859	2	3	2	2	2
Sample863	2	3	2	2	2
Sample882	2	3	2	2	2
Sample886	2	3	2	2	2
Sample905	2	3	2	2	2
Sample925	2	3	2	2	2
Sample933	2	3	2	2	2
Sample952	2	3	2	2	2
Sample991	2	3	2	2	2
Sample1063	2	3	2	2	2
Sample1071	2	3	2	2	2
Sample1083	2	3	2	2	2
Sample1097	2	3	2	2	2
Sample1135	2	3	2	2	2
Sample1142	2	3	2	2	2
Sample1153	2	3	2	2	2
Sample1194	2	3	2	2	2
Sample1222	2	3	2	2	2
Sample189	2	4	1	2	2
Sample129	2	4	2	1	2
Sample348	2	4	2	1	2
Sample772	2	4	2	1	2

Sample594	2	4	2	2	1
Sample155	2	4	2	2	2
Sample176	2	4	2	2	2
Sample556	2	4	2	2	2
Sample567	2	4	2	2	2
Sample750	2	4	2	2	2
Sample832	2	4	2	2	2
Sample1020	2	4	2	2	2
Sample560	2	5	2	1	2
Sample679	3	2	2	1	2
Sample728	3	2	2	2	2
Sample730	3	2	2	2	2

Low copy number, CN < 2; normal copy number, CN = 2; and high copy number, CN > 2.

Table S7. Copy number variants associated with the risk of gout in subgroups of gender

CNV region		CN < 2	CN = 2	CN > 2	*P**	$P^{\#}$
Male						
ABCF1	Case	9	479	14	0.007	0.101
	Control	10	586	3		
IL17REL	Case	0	499	3	0.002	1.85e-4
	Control	8	591	0		
FCGR3A	Case	8	454	40	0.042	0.028
	Control	8	516	75		
Female						
ABCF1	Case	1	25	0	0.612	0.457
	Control	2	96	1		
IL17REL	Case	1	25	0	0.506	0.568
	Control	2	97	0		
FCGR3A	Case	0	24	2	1.000	0.968
	Control	0	91	8		

**P* values were calculated by Fisher's exact test. $P^{\#}$ means *P* values were calculated by deviance analysis for logistic regression model after adjusted for age. CN is the abbreviation of copy number. CN = 2 was treated as referent group.
