# Supplementary material for: Copy number variants of ABCF1, IL17REL, and FCGR3A are associated with the risk of gout
Source: Protein Cell. 2017 Apr 12;8(6):467–70. doi: 10.1007/s13238-017-0401-y (PMC5445030; doi:10.1007/s13238-017-0401-y)
Supplement: Supplementary file 2 — Supplementary material 2 (PDF 97 kb) [file 13238_2017_401_MOESM2_ESM.pdf]

Study subjects

The present study was approved by the Ethical Committees of the School of Life Sciences of Fudan University. Informed consent form was signed by each participant in this study. A total of 626 gout patients and 18 other patients with rheumatic disorders (5 ankylosing spondylitis (AS) patients, 7 systemic lupus erythematosus (SLE) patients and 6 systemic sclerosis (SSc) patients) were recruited from Changhai Hospital, Taixing People’s Hospital and Taizhou People’s Hospital. Those gout patients had been clinically diagnosed with primary gout according to the American College of Rheumatology diagnostic criteria (Wallace et al., 1977). As a control population, 745 participants without history of gout, metabolic syndrome and other rheumatic diseases were recruited from Taizhou Longitudinal Study (Wang et al., 2009). Characteristics of the participants in this study were shown in S.Table 3 and 4.

DNA extraction

Peripheral blood was collected from all participants enrolled in this study. Genomic DNAs were isolated from whole blood using QIAamp DNA Blood Mini kit (QIAGEN, Germany) and then stored at -20℃immediately. The concentration and quality of DNAs (including optical density (OD) 260/280 and 260/230 measurements) was determined by a Nanodrop Lite spectrophotometer (Thermo Fisher’s Scientific, Waltham, MA, USA).

Genome-wide CNVs scanning: stage 1

The genomic DNAs from 22 patients with 4 types of rheumatic disorders were processed on the Agilent platform. Agilent SurePrint G3 Human 1 x 1 M comparative genomic hybridization (CGH) microarray (Agilent Technologies, Santa Clara, CA, USA) was used to

investigate the CNVs across the entire genome (Chen et al., 2014). Commercial male and female genomic DNAs (Promega, Madison, WI, USA) were used as gender-matched controls. The genomic DNAs extracted from the patients and the gender-matched controls were fragmented using the *Alu*I and *Rsa*I restriction enzymes. DNA labeling was performed using the Agilent SureTag DNA Labeling Kit. Different fluorescence dyes were used to label the DNA from the patients (Cy5-dUTP) and the controls (Cy3-dUTP). The labeled DNA from each patient was hybridized with the labeled gender-matched control DNA onto the Agilent SurePrint G3 Human 1 × 1 M microarray for 40 hours at 65°C. DNA processing, microarray handling and scanning were conducted according to the Agilent oligonucleotide CGH protocol (version 6.0).

The microarray scanning profiles were processed by Agilent Feature Extraction 10.7.3.1. The extracted data were analyzed and plotted by Agilent Workbench 7.0. ADM-2 was selected as the statistical algorithm, with the threshold of 6.0 and the Fuzzy Zero turned on. Each CNV was analyzed by at least five consecutive probes, with a log_2Ratio (fluorescence value ratio of gout sample-associated Cy5 to gender-matched control-associated Cy3) consistent with a specific value. The log_2Ratio of approximately 0.6 or 1 indicated a high copy number ($CN > 2$), while the log_2Ratio at approximately -1 indicated a low copy number ($CN < 2$). The genes present in the selected CNV regions were identified through the UCSC human genome browser based on the GRCh37/hg19 assembly.

Identification of gout-associated CNVs: stage 2

In this stage, a high-density CGH assay, designed and made by Roche, was used to identify CNVs in 48 pre-selected regions (filtered according to the frequency (> 5% and ≤ 50%), the genome location (in gene region), the gene function (related with inflammation and immunity), the reliability (at least five consecutive probes) and previous studies for these CNVs). A total of 46 gout patients were examined using the NimbleGen CGH Arrays (12 x 270K) according to the manufacturer's protocol. Pooled genomic DNAs from 47 healthy subjects (containing 27 men and 20 women) were used as the internal controls. Different fluorescence dyes were used to label the DNAs from the gout patients (ULS-Cy5) and the internal controls (ULS-Cy3). The labeled DNA from each patient was hybridized with the labeled control DNA onto the NimbleGen CGH Arrays for 60-72 hours at 42°C. Then, the two-color NimbleGen arrays were washed and then scanned with the NimbleGen MS 200 microarray scanner. The data were extracted using the MS 200 Data Collection Software.

The Log2 value, the median of the log_2Ratio, was extracted from the Roche platform raw data file. To protect the statistical models in the presence of outliers (e.g., probe failure), the Log2 values were converted to normal scores. A dynamic programming algorithm was employed to minimize the sum of the within segment variance minus a penalty term on the total number of segments. Finally, the genes and DGV CNVs that overlap with the identified CNVs were counted and annotated.

Gout-associated CNVs validation: stage 3

After further filtered by the frequency (> 5%) and the genome location (overlap with gene exon), five CNVs were tested in this stage using AccuCopy™. The AccuCopy™ technology

can scan the CNV status at multiple genomic loci in a single assay reaction (Wang et al., 2013). A total number of 1,274 participants (576 gout patients and 698 control subjects) were used to validate the gout-associated CNVs. All primer sequences for these CNVs were shown in S. Table 2. The AccuCopy assay was processed according to the manufacturer's protocol (Du et al., 2012). Briefly, four two-copy genes were selected as reference segments. The competitive double-stranded DNA sequence was synthesized for each target region and reference segment. Multiplex fluorescence competitive PCR was performed to simultaneously amplify all reference and target segments from both sample DNAs and competitive DNAs. The PCR products were diluted, separated by capillary electrophoresis, and the raw data were analyzed using GeneMapper 4.0 (ABI). The peak ratios between the sample and competitive DNAs (S/C) were calculated for all targeted regions and reference segments. The S/C ratio for each targeted region was normalized to the median of four reference segments, and then it was further normalized to the median value in all samples and finally averaged. The CN of each target region was tested by the average S/C ratio times two. The cases and controls were examined and analyzed at the same time to minimize non-random errors. The copy number (CN) was calculated by the comparative C_t method (Yu et al., 2011). Cut-off values of 0.25, 0.75 and 1.25 were used to define low copy number (CN $<$ 2), normal copy number (CN = 2) and high copy number (CN $>$ 2), respectively.

RNA isolation, cDNA synthesis, and real-time qPCR

The present study randomly collected RNA from 42 male gout patients and 46 healthy males. The RNAs were extracted from blood cells using TRIzol reagent according to the manufacturer's instructions (Invitrogen, Carlsbad, CA, USA). The complementary DNAs

(cDNAs) were synthesized through RNA reverse transcription using the High Capacity cDNA Reverse Transcription Kit (Applied Biosystems, Foster City, CA, USA) according to the manufacturer's protocol. Real-time quantitative polymerase chain reaction (qPCR) was performed using SYBR Premix Ex Taq (TaKaRa Biotech, Tokyo, Japan) with an ABI Prism 7900 Detector System (Applied Biosystems). The data obtained from the assays were analyzed by SDS 2.3 software (Applied Biosystems). The human housekeeping gene glyceraldehyde-3-phosphate dehydrogenase (*GADPH*) was used as an internal control.

Statistical analysis

Fisher's exact test and deviance analysis for the logistic regression model adjusted for age and gender were used to determine the statistically significant differences in the distribution of the copy numbers between the gout patients and control subjects. The Benjamini & Hochberg (BH) method was used to correct for multiple comparisons (Benjamini and Hochberg, 1995). The mRNA levels were illustrated as boxplots, and the outliers were removed. The differences in the mRNA expression of the candidate genes between the gout patients and healthy controls were analyzed by Student's *t*-test. In addition, we also queried two existing expression quantitative trait locus (eQTL) databases (Geuvadis data browser (http://www.ebi.ac.uk/Tools/geuvadis-das/) (Lappalainen et al., 2013) and Genotype-Tissue Expression Data Portal (http://www.gtexportal.org/home/)) (Lonsdale et al., 2013) to analyze the association of eQTL in candidate CNVs with transcript expression. A *P* value less than 0.05 was considered statistically significant. All statistical analysis was performed using R (Version 3.0.2: www.r-project.org/).

References:

Benjamini, Y., and Hochberg, Y. (1995). Controlling the False Discovery Rate - a Practical and Powerful Approach to Multiple Testing. Journal of the Royal Statistical Society Series B-Methodological 57, 289-300.

Chen, Y., Guo, L., Chen, J., Zhao, X., Zhou, W., Zhang, C., Wang, J., Jin, L., Pei, D., and Zhang, F. (2014). Genome-wide CNV analysis in mouse induced pluripotent stem cells reveals dosage effect of pluripotent factors on genome integrity. BMC Genomics 15, 79.

Du, R.Q., Lu, C.C., Jiang, Z.W., Li, S.L., Ma, R.X., An, H.J., Xu, M.F., An, Y., Xia, Y.K., Jin, L., *et al.* (2012). Efficient typing of copy number variations in a segmental duplication-mediated rearrangement hotspot using multiplex competitive amplification. Journal of Human Genetics 57, 545-551.

Lappalainen, T., Sammeth, M., Friedlander, M.R., t Hoen, P.A., Monlong, J., Rivas, M.A., Gonzalez-Porta, M., Kurbatova, N., Griebel, T., Ferreira, P.G., *et al.* (2013). Transcriptome and genome sequencing uncovers functional variation in humans. Nature 501, 506-511.

Lonsdale, J., Thomas, J., Salvatore, M., Phillips, R., Lo, E., Shad, S., Hasz, R., Walters, G., Garcia, F., Young, N., *et al.* (2013). The Genotype-Tissue Expression (GTEx) project. Nature Genetics 45, 580-585.

Wallace, S.L., Robinson, H., Masi, A.T., Decker, J.L., McCarty, D.J., and Yu, T.F. (1977). Preliminary criteria for the classification of the acute arthritis of primary gout. Arthritis Rheum 20, 895-900.

Wang, J., Yang, Y., Guo, S., Chen, Y., Yang, C., Ji, H., Song, X., Zhang, F., Jiang, Z., Ma, Y., *et al.* (2013). Association between copy number variations of HLA-DQA1 and ankylosing spondylitis in the Chinese Han population. Genes and Immunity 14, 500-503.

Wang, X.F., Lu, M., Qian, J., Yang, Y.J., Li, S.L., Lu, D.R., Yu, S.Z., Meng, W., Ye, W.M., and Jin, L. (2009). Rationales, design and recruitment of the Taizhou Longitudinal Study. BMC Public Health 9.

Yu, B., Guan, M., Peng, Y., Shao, Y., Zhang, C., Yue, X., Zhang, J., Yang, H., Zou, H., Ye, W., *et al.* (2011). Copy number variations of interleukin-17F, interleukin-21, and interleukin-22 are associated with systemic lupus erythematosus. Arthritis Rheum 63, 3487-3492.
